# Growth arrest specific gene 7 is associated with schizophrenia and regulates neuronal migration and morphogenesis

**DOI:** 10.1186/s13041-016-0238-y

**Published:** 2016-05-18

**Authors:** Zhengrong Zhang, Fanfan Zheng, Yang You, Yuanlin Ma, Tianlan Lu, Weihua Yue, Dai Zhang

**Affiliations:** Institute of Mental Health, The Sixth Hospital, Peking University, 51 Hua Yuan Bei Road, Hai Dian District, Beijing, 100191 China; Key Laboratory of Mental Health, Ministry of Health & National Clinical Research Center for Mental Disorders (Peking University), Beijing, 100191 China; Brainnetome Center, Institute of Automation, Chinese Academy of Sciences, 95 Zhong Guan Cun East Road, Hai Dian District, Beijing, 100190 China; Peking-Tsinghua Center for Life Sciences, Peking University, Beijing, 100871 China; PKU-IDG/McGovern Institute for Brain Research, Peking University, Beijing, 100871 China

**Keywords:** Schizophrenia, *GAS7*, Neurite outgrowth, Neuronal migration

## Abstract

**Background:**

Schizophrenia is a highly heritable chronic mental disorder with significant abnormalities in brain function. The neurodevelopmental hypothesis proposes that schizophrenia originates in the prenatal period due to impairments in neuronal developmental processes such as migration and arborization, leading to abnormal brain maturation. Previous studies have identified multiple promising candidate genes that drive functions in neurodevelopment and are associated with schizophrenia. However, the molecular mechanisms of how they exert effects on the pathophysiology of schizophrenia remain largely unknown.

**Results:**

In our research, we identified growth arrest specific gene 7 (*GAS7*) as a schizophrenia risk gene in two independent Han Chinese populations using a two-stage association study. Functional experiments were done to further explore the underlying mechanisms of the role of Gas7 in cortical development. In vitro, we discovered that Gas7 contributed to neurite outgrowth through the F-BAR domain. In vivo, overexpression of Gas7 arrested neuronal migration by increasing leading process branching, while suppression of Gas7 could inhibit neuronal migration by lengthening leading processes. Through a series of behavioral tests, we also found that Gas7-deficient mice showed sensorimotor gating deficits.

**Conclusions:**

Our results demonstrate *GAS7* as a susceptibility gene for schizophrenia. Gas7 might participate in the pathogenesis of schizophrenia by regulating neurite outgrowth and neuronal migration through its C-terminal F-BAR domain. The impaired pre-pulse inhibition (PPI) of Gas7-deficient mice might mirror the disease-related behavior in schizophrenia.

**Electronic supplementary material:**

The online version of this article (doi:10.1186/s13041-016-0238-y) contains supplementary material, which is available to authorized users.

## Background

Schizophrenia is a multifactorial neurodevelopmental disorder with a prevalence of about 1 %. To date, genome-wide association studies have revealed hundreds of genes, which are involved in neuronal calcium signaling, synaptic plasticity and neurodevelopment, as schizophrenia susceptibility genes [1]. However, the underlying mechanisms of how these genes contribute to schizophrenia remain elusive. Biological experiments are necessary to further explore the molecular underpinnings of these genes.

The precise function of the brain relies on the complex architecture of neural circuits that are established during development. Defects in neuronal morphogenesis and cell migration in the cerebral cortex can cause disruptions in neural circuits that lead to mental disorders including schizophrenia [2]. Microtubules and actin are essential components of the cytoskeleton that play critical roles in neurodevelopment and shaping neural networks [3, 4]. The basis of neurite initiation, outgrowth and branching is rooted in the ability of the actin and microtubule cytoskeleton to undergo dynamic changes [5–7]. In developing neurons, microtubules steer growth cones as they interact with actin filaments contributing to growth cone advance and turning in filopodia, and consequently govern axon guidance and synaptic plasticity [8]. Abnormalities in those processes may alter the strength of information processing and thus participate in the pathogenesis of human developmental brain diseases such as schizophrenia [9]. A recent paper reported that, in cultured olfactory neuroepithelial cells from individuals with schizophrenia, the stability of microtubules was increased [10].

Growth arrest specific gene 7 (*GAS7*) was initial identified in growth-arrested NIH3T3 fibroblasts [11, 12]. Previous studies have demonstrated that Gas7 is abundantly expressed in the central nervous system (CNS), including the cerebral cortex, hippocampus and cerebellum [13]. In addition, functional studies have indicated that Gas7 is required for neurite outgrowth and neuronal differentiation [13–15]. The Gas7 protein can directly interact with F-actin to enhance actin polymerization [16]. Recent studies have reported a novel role for Gas7 in maintaining microtubule stability and polymerization [17]. In light of these findings, we hypothesized that Gas7 might play a role in the pathogenesis of schizophrenia and deficits in Gas7 might impact normal brain development.

To date, there is no direct evidence revealing the biological function of Gas7 during brain development, although the importance of Gas7 during neurite outgrowth and neuronal differentiation has emerged recently [13, 15]. In the present study, we first conducted a two-stage case control association study in two independent Han Chinese populations. We found that *GAS7* is significantly associated with schizophrenia. We performed in vitro and in vivo assays to explore the biological functions of Gas7 during brain development. We found that Gas7 is involved in neurite outgrowth and neuronal migration through its F-BAR domain. In addition, we focused on schizophrenia-related endophenotypes that are reliably modeled in mice. Our results identify *GAS7* as a susceptibility gene for schizophrenia and highlight the functional importance of proteins directly regulating membrane deformation for proper neuronal migration and morphogenesis.

## Results

### Positive association of *GAS7* and schizophrenia

We performed a two-stage association study of schizophrenia (SZ) in the Han Chinese population. In stage one, we screened seven single nucleotide polymorphisms (SNPs) in *GAS7* from our initial genome-wide association study (GWAS) data [18], which detected three associated SNPs, rs9908211, rs12150284 and rs11656696 (Table 1). In stage two, an independent population, including 2514 unrelated schizophrenia patients (1128 males and 1386 females; mean age: 32.4 ± 8.6 years) and 2637 healthy control subjects (1187 males and 1450 females; mean age: 31.8 ± 9.3 years), was recruited for validation. The results support the associations of all three SNPs with SZ (Table 1).

**Table 1:** The summary results of single marker association for the SNPs of *GAS7* gene in two-stage study

Stage	Marker	Associated Allele	Frequency in Case	Frequency in Control	OR (95%CI)	*P* value
1	rs12450747	A	0.464	0.496	0.88 (0.78–0.99)	0.036
	rs11649731	A	0.585	0.558	1.12 (0.99–1.27)	0.073
	rs12452356	A	0.876	0.895	0.83 (0.69–1.01)	0.056
	rs9908211	A	0.268	0.222	1.28 (1.11–1.47)	6.98E-4
	rs12150284	C	0.504	0.450	1.24 (1.10–1.40)	5.90E-4
	rs11656696	A	0.481	0.533	0.81 (0.72–0.92)	7.81E-4
	rs7208708	C	0.506	0.547	0.85 (0.75–0.96)	0.0078
2	rs9908211	A	0.246	0.225	1.12 (1.03–1.23)	0.012
	rs12150284	C	0.487	0.452	1.15 (1.06–1.24)	4.20E-4
	rs11656696	A	0.494	0.532	0.86 (0.80–0.93)	1.69E-4

All of the seven SNPs we selected showed minor allele frequencies (MAFs) greater than 5 % in our samples. The genotype distributions of the seven SNPs in either the case or the control groups did not show significant deviations from Hardy-Weinberg equilibrium (HWE) (*P >* 0.05). No significant differences in age or gender distributions were found between the case and control samples. The allele frequencies of the seven SNPs are shown in Table 1.

Given that all the positive SNPs are located in the intron region of the genome and previous results from GWASs have indicated that the functions of associated intron SNPs exert their effects through altering gene expression rather than protein structure [19, 20], we decided to investigate the functions of *GAS7* by overexpression and knockdown assays with in vitro and in vivo approaches.

### Both overexpression and knockdown of Gas7 result in aberrant neuronal morphogenesis

**Fig. 1:**
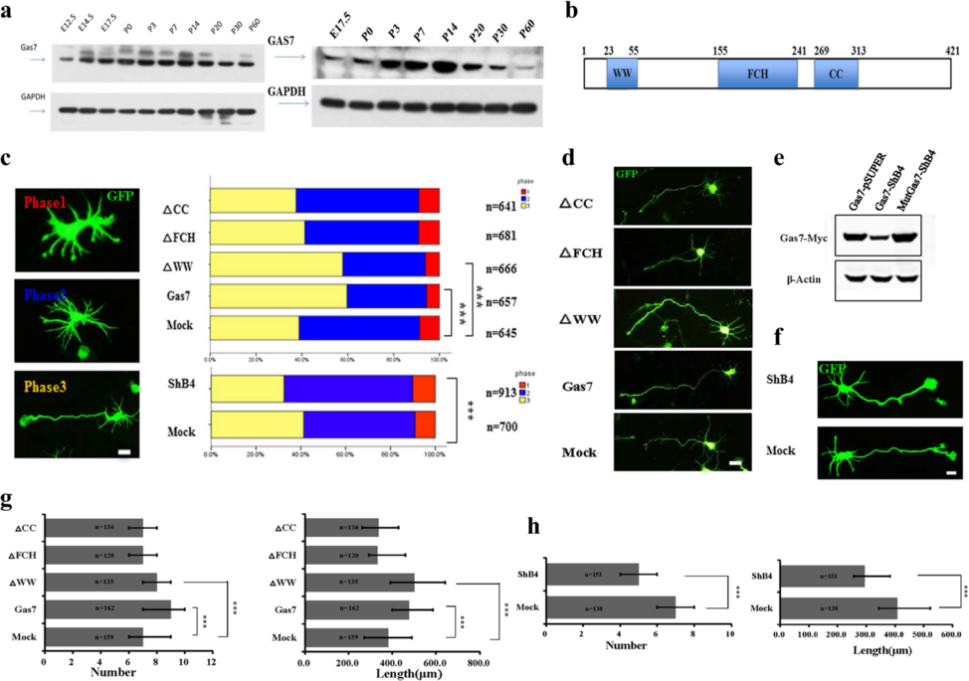
Gas7 influenced development stages of cortical neurons through F-BAR domain in vitro. **a** Immunoblotting revealed the Gas7 levels in the cerebral cortex (*left*) and hippocampus (*right*) during development. **b** Schematic representation of domain organization of Gas7. **c** Morphological characterization of cortical neurons in early three stages (*left*). Percentage of cortical neurons transfected with Gas7, pCAG-IRES vector (Mock) and truncates of Gas7 (△CC, △FCH, △WW) in different stages (*up row*). Percentage of cortical neurons transfected with pSUPER (Mock) and shRNAs target-Gas7 (ShB4) in different stages (below row). **d** Representative images of cortical neurons at stage-3, which transfected at DIV0 for 3d with different truncates of Gas7, Gas7 and Mock by electroporated transfection. **e** Immunoblotting in HEK293T cells showed that Gas7 specific shRNAs, ShB4 is efficient to knockdown Gas7 rather than shRNA-resistant Gas7 (synonymous mutation). **f** Representative images of cortical neurons at stage-3 which transfected at DIV0 for 3d with mock, ShB4 together with GFP. **g** Quantification of number of the primary neurites (*left*) and the length of the longest neurite (*right*) as shown in Fig. 1d. Data represent mean ± SEM. *N* = 3 (number as indicated) for each group. **h** Quantification of number of the primary neurites (left) and the length of the longest neurite (*right*) as shown in Fig. 1f. Data represent mean ± SEM. *N* = 3 (number as indicated) for each group. Scale bar, 5 μm

**Fig. 2:**
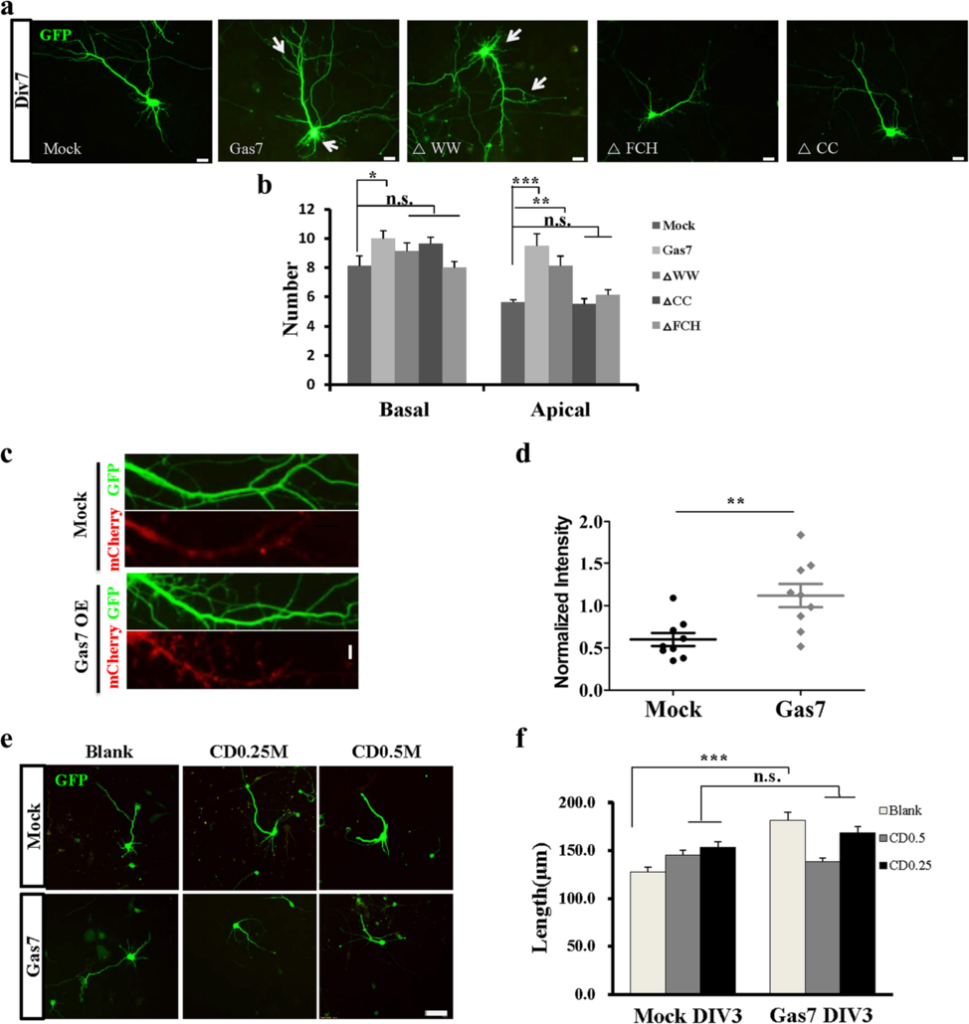
Overexpression of Gas7 and △WW can contribute to aberrant neuron morphogenesis by inducing filopodia. **a** Representative images of cortical neurons transfected at DIV 4 for 3 d with different truncates of Gas7 (△CC, △FCH, △WW), Gas7, pCAG-IRES (Mock) by calcium phosphate transfection. Scale bar, 20 μm. **b** Quantification of numbers of basal and apical dendrite branch as shown in Fig. 2a. Data represent mean ± SEM. *N* = 3 (eight neurons) for each group. **c** Gas7 regulated F-actin and contributed to F-actin assembly. Plasmids were co-transfected with mCherry-UtrCH, a versatile fluorescent probe for actin filaments. Scale bar, 5 μm. **d** Quantification of normalized intensity as shown in Fig. 2c. Data represent mean ± SEM. *N* = 3 (9 neuron) for each group, two fragments per neuron and GFP fluorescence intensity as control. **e** Representative images of cortical neurons transfected with Mock and Gas7 at DIV 0 for 3d, which were acute treated with Cytochalasin D (30 min). **f** Quantification of length of the longest neurite as shown in Fig. 2e. Data represent mean ± SEM. *N* = 3 (>100 neuron) for each group. Scale bar, 50 μm

We began our study of the role of Gas7 in neuronal development by identifying the function of Gas7 during early neuronal development in mice. First, we measured the abundance of Gas7 in the developmental cerebral cortex and hippocampus. The peak level of Gas7 was present in the cortex and hippocampus at P7 and P14 (Fig. 1a). We then transfected neurons at 0 days in vitro (DIV0) with Gas7 plasmid and observed the morphology of neurons according to previous methods (Fig. 1c) [21, 22]. As early as DIV3, more neurons transfected with Gas7 were found to develop into stage 3, compared with EGFP-transfected controls (Fig. 1c). We calculated the number of dendrites (the primary neurites) and the length of the axon (the longest neurite) (Additional file 1: Figure S1A). The average number of primary neurites was higher, as well as the length of the longest neurite (Fig. 1d and g). We next tested the function of Gas7 in neuronal morphogenesis by designing short hairpin interfering RNA (shRNA) and silent mutant as a shRNA-resistant form of Gas7, to acutely knockdown Gas7 expression and exclude the possibility of off-target effects, respectively (Fig. 1e). As expected, neurons transfected with shRNA showed the opposite effect compared with overexpression, as a higher percentage of the neurons stayed in stage 1 and stage 2 (Fig. 1c), and neurons developing into stage 3 exhibited less primary neurites and shorter axons (Fig. 1f and h). To study the effect of Gas7 in later stages, we transfected neurons using calcium phosphate at DIV4 and fixed the cells at different developmental stages. As early as DIV7, overexpression of Gas7 caused overgrowth of both apical dendrites and basal dendrites, mainly evidenced by increased numbers of primary and secondary dendrites compared with controls (Fig. 2a and b). As late as DIV10, these excessive dendrites kept growing and became net-shaped around the cell body, and by DIV21 we could not distinguish individual dendrites (Additional file 2: Figure S2A). The neurons with aberrant morphogenesis were EMX1-positive, indicating that they were cortical pyramidal neurons (Additional file 2: Figure S2B). When neurons were transfected with shRNA, significant reductions in both axonal and dendritic branching were observed at DIV7. This effect could be rescued by co-transfection of a shRNA-resistant form of Gas7, demonstrating that this is not an off-target effect (Additional file 2: Figure S2C). The fact that changes in Gas7 expression level can influence branching in cortical neurons, a process previously shown to require filopodia formation, suggests that Gas7 promotes neurite branching through its ability to induce filopodia in neurons [23, 24].

### Both full-length Gas7 and its F-BAR domain induce filopodia formation

It is generally accepted that Gas7 possesses three functional domains: the N-terminal WW domain, the middle Fes/CIP4 homology (FCH) domain and the C-terminal coiled-coil (CC) domain (Fig. 1b). The FCH domain and the CC region have also been represented as a single extended domain, the F-BAR domain, which mediates oligomer formation and interactions with the submembrane region [25]. Overexpression of Gas7 can induce filopodia formation (Additional file 3: Figure S3A, B). Furthermore, expression of the isolated F-BAR domain (Gas7^△WW^) also induced filopodia formation, suggesting that the F-BAR domain maybe be a membrane-targeting motif (Additional file 3: Figure S3C). This effect requires the F-BAR domain because deletion of the CC (Gas7^△CC^) or FCH (Gas7^△FCH^) domain does not induce filopodia formation in SH-SY5Y cells (Additional file 3: Figure S3D, E). These data suggest that the F-BAR domain of Gas7 is necessary for induction of filopodia-like membrane protrusions (Additional file 3: Figure S3F).

### Gas7 regulates neurite formation and branching through the ability of its F-BAR domain to form filopodia

To further identify the functions of each domain in neuronal morphogenesis, we constructed truncates of Gas7 and transfected neurons with them. Indeed, we found that Gas7^△WW^-overexpressing neurons displayed similar morphogenesis to those overexpressing full-length Gas7, while overexpression of Gas7^△FCH^ or Gas7^△CC^ had no effect on the ability of neurons to initiate excessive neurites (Figs. 1d, g and 2a, b), suggesting that the F-BAR domain is necessary for normal function of Gas7 and the ability of the F-BAR domain to induce filopodia requires the existence of both the FCH and CC domains. According to previous research, the C-terminal of Gas7 can directly interact with actin and cause F-actin assembly [16]. This is confirmed by further experiments where we co-transfected neurons with control or *Gas7* plasmid with mCherry-UtrCH (a visible red probe of F-actin) [26], and found that the F-actin, which was tagged with mCherry and immunostained as a red color, is co-localized with Gas7 (Additional file 4: Figure S4A). Statistical analysis showed that neurons transfected with *Gas7* exhibited F-actin assembly with more dense red fluorescent intensity compared with control neurons (Fig. 2c and d). To investigate if there are other underlying mechanisms, we added Cytochalasin D to the culture medium. After Cytochalasin D treatment, we found that overexpression of full-length Gas7 no longer promoted the development of neurons (Fig. 2e and f). These data suggest that the F-BAR domain of Gas7 can regulate neurite formation and branching through interaction with actin. Together, these data suggest that Gas7 increases neurite initiation and branching through the ability of its F-BAR domain in mediating F-actin polymerization to induce filopodia.

### Overexpression of Gas7 inhibits radial migration of cortical pyramidal neurons by increasing leading process branching

**Fig. 3:**
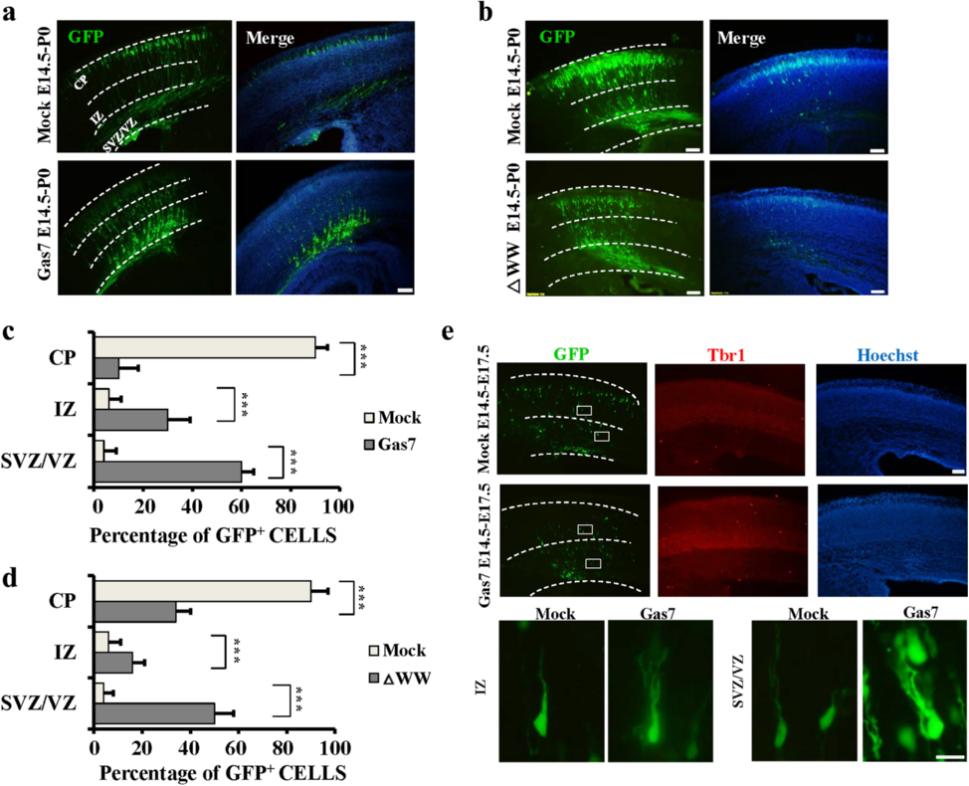
Overexpression of Gas7 inhibited radial migration of cortical pyramidal neurons by increasing leading process branching. **a**, **b** Representative images showing the E14.5 mouse cortices electroporated with overexpress Gas7, Gas7 ^△WW^ and pCAG-IRES vector (Mock) and examined at P0. Representative coronal brain sections at P0 were stained with antibodies to GFP (*green*) and counter stained with Hoechst (blue). Scale bar, 100 μm. **c**, **d** Numbers of GFP-positive neurons is quantified in SVZ/VZ, IZ and CP zones of brain. Data represent mean ± SEM. *N* = 4 for each group. **e** Representative images showing the E14.5 mouse cortices electroporated with indicated Gas7 or mock plasmid and examined at E17.5. Representative coronal brain sections were stained with antibodies to GFP (*green*), Tbr1 (a 5-layer marker, *red*) and Hoechst (*blue*). Enlarged view of electroporated neurons show excessive LP branching in the IZ and SVZ/VZ zones. Scale bars, 100 μm (*up row*) and 5 μm (*below row*)

During brain development, neuronal migration requires considerable changes in cell shape involving coordinated cytoskeletal and membrane remodeling [27]. Neuronal migration involves the coordinated extension and adhesion of the leading process (LP) along the radial glial scaffold [27]. The tremendous changes in neuronal morphogenesis after overexpression of Gas7 made us wonder if the neurons can migrate to the target layer of the cerebral cortex. To determine the effects of Gas7 and the F-BAR domain during cortical development, we introduced our truncated constructs into radial glial progenitors at embryonic day 14.5 (E14.5) using in vivo electroporation. We hypothesized that overexpression of Gas7 or its F-BAR domain should be sufficient to block migration by increasing filopodia formation. At postnatal day 0 (P0), we found that overexpression of Gas7 and the F-BAR domain truncate (Gas7^△WW^) severely inhibited radial migration compared with that in control EGFP-expressing slices (Fig. 3a and b). We quantified radial migration by determining the ratio of neurons in the Cortical Plate (CP, where pyramidal neurons complete migration), intermediate zone (IZ, where they initiate radial migration) and ventricular/subventricular zone (VZ/SVZ, where the majority of neurons are generated). The percentage of neurons migrated to the CP is significantly decreased by Gas7 or F-BAR domain truncate overexpression (Fig. 3c and d). In contrast, the overexpression of Gas7^△FCH^ or Gas7^△CC^ had no effect on neuronal migration compared with that in cells transfected with control EGFP plasmids (Additional file 5: Figure S5A, B). To explore the mechanism underlying this migration delay, we dissected embryonic mice brains at E17.5, when most of the cortical pyramidal neurons are still on their way to the cortical plate [27]. The LPs of full-length Gas7 transfected neurons displayed excessive branches (Fig. 3e). This is consistent with previous research reporting that excessive LP branching in migrating cortical neurons can inhibit neuronal migration [28, 29]. Altogether, these data suggest that Gas7 increases neurite initiation and branching through the ability of its F-BAR domain to induce filopodia, which in turn negatively regulates neuronal migration.

### Reduction of Gas7 expression affects neuronal migration by extending leading process length

**Fig. 4:**
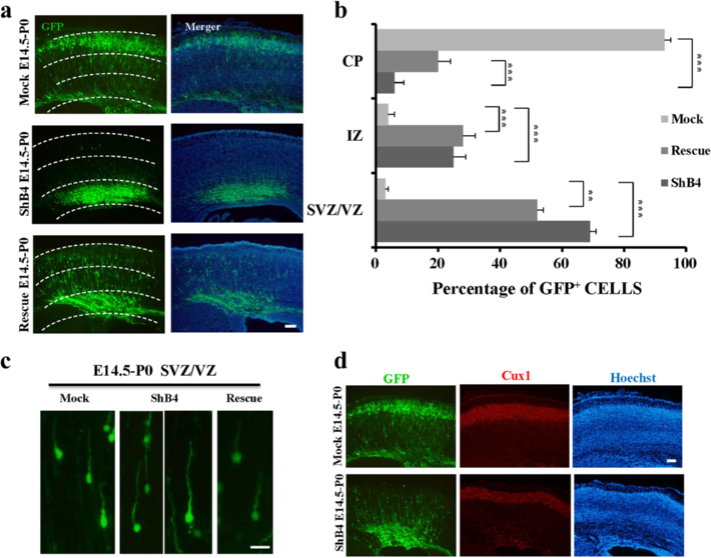
Reduction of Gas7 expression affects neuronal migration by extending leading process length. **a** Representative images showing the E14.5 mouse cortices electroporated with GFP plasmids together with pSUPER vector (Mock), shRNAs targeting Gas7 (ShB4) and shRNA-resistant Gas7 (Rescue) and examined at P0. Majority of transfected neuron by ShB4 were in the IZ or SVZ zones. Migration defect can be partly rescued. Scale bar, 100 μm. **b** Numbers of GFP-positive neurons is quantified in three brain zones. Data represent mean ± SEM. *N* = 4 for each group. **c** Enlarged view of electroporated neurons after depletion of Gas7 show extending LP length, while it can be recused by shRNA-resistant Gas7. Scale bar, 5 μm. **d** In utero electroporation at E14.5 with ShB4 and Mock as indicated. Representative coronal brain sections were stained with antibodies to GFP (green), Cux1 (a 2–3 layer marker, *red*). Scale bar, 100 μm

We next introduced Gas7 shRNA into E14.5 cortical neurons to examine the effect of Gas7 knockdown during brain development. Previous research reported that a decrease in LP branching can promote neuronal migration [30]. We hypothesized that, Gas7 knockdown might reduce LP branching and promote migration, considering the opposite effect that was observed on neuronal morphogenesis. To our surprise, at P0, we found that slices expressing shRNA showed a significant decrease in the percentage of neurons that have reached the CP and a corresponding increased percentage of neurons in the IZ. This suggested that reduction of Gas7 expression also decelerated radial migration and this inhibition might be partly rescued (Fig. 4a and b). All the delayed neurons express Cux-1, a layer 2–3 specific neuronal marker, indicating that disruption of Gas7 did not affect neuronal differentiation (Fig. 4d). To understand the mechanism underlying this phenomenon, we analyzed single cell morphology in dissected embryonic mice brains. We observed that, neurons expressing shRNA demonstrated an elongated LP compared with neurons expressing control EGFP plasmids (Fig. 4c), which might be a manifestation of in vivo compensatory mechanisms of the decreased neurite branching we observed in cultured neurons in vitro. Given that neuronal migration requires a series of delicately coordinated of biological processes [27], we speculate that the elongated LP cannot complete the proper extension and adhesion required for migration and this leads to the delayed development of neurons.

### Gas7-deficient mice display impaired pre-pulse inhibition

**Fig. 5:**
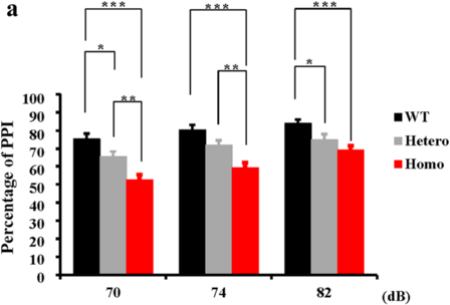
Behavior tests of Gas7 deficient mice. **a** The Gas7 deficient mice performed abnormal in pre-pulse inhibition. Homozygous mice showed a significant reduction in the ability to gate the startle response (to 120 dB), as indicated by decreased pre-pulse inhibition at three kinds of startle (70, 74, 82 dB). The heterozygous mice showed moderate deficits in PPI. WT, *N* = 12; Hetero, *N* = 9; Homo, *N* = 15

Considering all the effects observed above, we wondered whether changes in Gas7 expression level would induce schizophrenia-like behaviors. We tested a previously constructed Gas7-deficient mouse model [31] with a series of behavioral tests including sensorimotor gating (pre-pulse inhibition; PPI), psychomotor agitation (locomotor activity) and hippocampus-dependent learning and memory (Morris water maze) [32–34]. We first identified the expression pattern of Gas7 in this strain and found wild-type Gas7 was absent in both the hippocampus and cortex (Additional file 6: Figure S6A). During the behavioral tests, we found a significant dose-dependent effect of Gas7 expression on pre-pulse inhibition (PPI). A well-known and important intermediate phenotype of schizophrenia is impaired sensorimotor gating. Compared with wild type mice, the homogeneous mice showed the most severe impairment in PPI, while the heterogeneous mice showed a milder impairment (Fig. 5a). However, in the Morris water maze test, a test to monitor spatial working memory function, which is one of the most frequently impaired cognitive domains in patients with SZ [35], we found no significant difference between wild type and Gas7-deficient mice in latency to find the platform, although there was a slight trend for the mice to remain in the initial quadrant during the reverse phase (Additional file 6: Figure S6B–D). In addition, the Gas7-deficient mice displayed normal weight, locomotors activity and rotator test performance (Additional file 6: Figure S6E–G). We also excluded anxiety-like phenotypes in the deficient mice, by examining dark-light transition and open field in the first 10 min (Additional file 6: Figure S6H, I).

## Discussion

### *GAS7* is significantly associated with schizophrenia

Previous research demonstrated that Gas7 is highly expressed in cerebral cortex, hippocampus and cerebellum [13], all of which are reported to be key brain regions involved in the pathogenesis of schizophrenia. Recent studies on biological function of Gas7 revealed that this gene plays an important role in actin and microtubule polymerization [16, 17]. Considering that both actin and microtubules are essential in neural development and schizophrenia has been long considered a neurodevelopmental disease, we hypothesized that *GAS7* might be a susceptibility gene of schizophrenia and conducted a two-stage association study. Our results revealed that *GAS7* is significantly associated with schizophrenia. To further explore the potential mechanisms underlying this association, we employed in vitro and in vivo approaches to identify the roles of Gas7 during cortical development.

### A role for Gas7 during neuronal development

It is widely acknowledged that cytoskeletal dynamics produce forces to generate plasma membrane protrusions and invaginations; however, recent evidence suggests that many membrane-associated proteins directly sculpt and deform biological membranes [36]. Our study may suggest that Gas7 regulates neuronal migration as well as neurite initiation and branching, through the ability of its F-BAR domain to deform membranes and form filopodia-like membrane protrusions. This is quite a surprising result given that F-BAR domains have mostly been characterized for their ability to induce membrane tabulation and invaginations [37, 38]. In previous research, srGAP2 and PSTPIP2 have also been demonstrated to induce filopodia [30, 39, 40]. Notably, both GAS7 and PSTPIP2 belong to the Pombe Cdc15 homology (PCH) family involved in the regulation of actin-based functions and have been shown to directly regulate F-actin bundling and enhance filopodia formation [16, 40]. The F-BAR domain of srGAP2 has been shown to promote neurite initiation and branching, and this ability appears to be important for its regulation of migration [30]. The roles of Gas7 in neurite initiation and branching are likewise important for the regulation of migration.

Overexpression of Gas7 and srGAP2 can both induce excessive neurites and result in inhibition of migration. However, knockdown of Gas7 exhibited an opposite effect on radial migration compared with knockdown of srGAP2. Knockdown of srGAP2 significantly reduced LP complexity and branching and increased the rate of migration while knockdown of Gas7 increased LP length and decreased the rate of migration. Thus, both overexpression and knockdown of Gas7 can dramatically inhibit neuronal migration. The similarity of these neuronal migration defects may result from two different mechanisms. Overexpression of Gas7 could induce filopodia and significantly increase leading process complexity and branching. It has been well accepted that, when neurons migrate into IZ-SVZ, they transform from multipolar cells that are highly dynamic, extending and retracting processes to bipolar cells that extend a pia-directed leading process [41, 42]. Overexpression of Gas7 might result in a delay in that process which inhibits neuronal migration. However, we observed longer leading processes following knockdown of Gas7. That is, neurons could transform normally from multipolar to bipolar. But in the later phase, the elongated LP cannot make the proper extension and adhesion to radial glial guide fibers required for migration and this leads to inhibition of migration. Further study is needed to investigate the precise mechanisms underpinning the similarity of neuronal migration defects caused by manipulation of Gas7.

Our finding of abnormal neuron migration induced by in vivo electroporation of shRNA may seem inconsistent with previous work reporting no alteration in neuronal morphology in later developmental stages of Gas7-deficient mice [31]. However, it is quite understandable given that (1) the significant inhibition of neuronal migration during embryonic stage is a short-term response to the acute reduce of Gas7 level; (2) the Gas7-deficient mice express a labile Gas7 mutant protein whose functions are similar to wild-type Gas7 and the effects of losing wild-type Gas7 can be, at least in part, compensated by the functions of the mutant protein. Besides, there could be some other compensatory effects from upstream and downstream signaling pathways during the whole developmental stage of the mice and thus result in no alterations in neuronal morphology.

Previous research, most F-BAR domain-containing proteins possess SH3 domains [43], which can bind to effectors of actin polymerization such as WAVE-1. However, there is no SH3 domain in any of the isoforms of mouse Gas7 protein. An earlier study reported that the WW domain of Gas7 could interact with N-WASP [15]. However, we observed similar effects of both full length Gas7 and truncates without the WW domain on neurite branching and radial migration, indicating that this effect was not due to interaction with N-WASP, but might be through the direct interaction of F-actin and the F-BAR domain, as shown previously [16]. Future experiments should test how these two pathways function in balance to facilitate membrane protrusion.

### Gas7-deficiency induces schizophrenia-like behavior

Schizophrenia is a neurodevelopmental mental disorder characterized by psychotic symptoms and cognitive deficits, which include impaired working memory function and sensorimotor integration that can be evaluated in mice by the Morris water maze and pre-pulse inhibition (PPI) behavioral tests, respectively [44]. PPI is a measure of sensorimotor gating function. As indicated by a previous study, impairment in PPI has been recognized as an important intermediate phenotype for SZ [45]. In Gas7-deficient mice, although we did not find significant impaired working memory functions, we observed impaired PPI, indicating that the decreased Gas7 expression level might impact sensory motor gating function. It is worth noting that the Gas7-deficient mouse model was established by replacing Gas7 with a form of labile mutant protein, and the function of the mutant protein should not be ignored. We will try to construct knock-out mice, which might exhibit more schizophrenia-related behaviors. So, together with our genetic and functional study, the deficit in PPI could suggest that *GAS7* might be a risk gene for schizophrenia. Further investigation of the roles of Gas7 in the pathogenesis of schizophrenia is warranted.

## Conclusions

Our results suggest that *GAS7* is a candidate gene for schizophrenia. Gas7 plays a role in neuronal morphogenesis and migration, inducing filopodia-like membrane protrusions through its F-BAR domain. In addition, Gas7-deficient mice showed sensorimotor gating deficits, which could be a manifestation of schizophrenia-related behaviors. Our study highlights the functional importance of the susceptibility genes involved in neuronal development, and agrees with the neurodevelopmental hypothesis of schizophrenia.

## Methods

### Genome-wide association study

Our initial GWAS sample consisted of 768 unrelated subjects with SZ (360 males and 408 females) and 1348 control subjects (658 males and 690 females). For validation, an independent sample consisting of 1957 cases (1037 males and 920 females) and 1509 controls (360 males and 1149 females) was recruited. The consensus diagnoses were made by at least two experienced senior psychiatrists according to the Diagnosis and Statistical Manual of Mental Disorders Fourth Edition (DSM-IV) criteria for schizophrenia. Patients with severe medical complications were excluded. Healthy controls were recruited from communities with simple non-structured interviews performed by psychiatrists, who excluded individuals with histories of mental health and neurological diseases. All participants were unrelated Han Chinese recruited from the North of China. The study was approved by the Medical Research Ethics Committee of the Institute of Mental Health, Peking University. All participants enrolled in the study signed written informed consent.

Peripheral blood samples were collected from all subjects. Genomic DNA was extracted using the Qiagen QIAamp DNA Mini Kit (Qiagen, Germany). In the screening stage, we derived genotypes of seven SNPs in GAS7 from our GWAS data: rs12450747, rs11649731, rs12452356, rs9908211, rs12150284, rs11656696 and rs7208708. In the validation stage, the genotypes of rs9908211, rs12150284 and rs11656696 were determined using the Sequenom MassARRAY system (Sequenom iPLEX). 1957 cases and 1059 controls were successfully genotyped; the calling rate being 99.91 %.

### Construction of plasmids

Mouse Gas7 plasmid was gifted from Prof. Sue Lin-Chao and truncates were generated according to the functional domains of the schematic representation from previous research [15]. The plasmids expressing full Gas7 or truncates were fused with MYC at C-terminal and then subcloned to pCAGGS-IRES-EGFP vector. CAG-enhanced green fluorescent protein (EGFP) was used as the control. shRNAs targeting Gas7 oligonucleotides were inserted into pSUPER. The sequences were as follows: GGCCCAGTCCAAGTGGTTTGA. shRNA-resistant sequences were GGCACAATCAAAATGGTTCGA. One nucleotide in the codons was mutated in Gas7 without altering the identity of amino acids. Venus EGFP was co-transfected with pSUPER for analytical purposes.

### Primary neuronal culture

Cortical neurons of E17.5 ICR fetuses (from Department of Laboratory Animal Science, Peking University Health Science Center) were isolated in HBSS (Invitrogen) at 4 °C. Neurons were plated on poly-d-lysine and laminin pre-coated glass coverslips in Neurobasal/B27 medium, maintained in culture and then fixed at different time points for immunofluorescence analysis.

### Transfection

HEK293T cells were cultured in DMEM containing 10 % fetal bovine serum, and were transfected in a 35 mm dish with 2 μg plasmid (Gas7: ShB4, 3:1) and 5 μl Lipofectamine 2000 (Invitrogen) in serum-free medium for 6 h, and then cultured in medium containing serum for 2 days. Cortical neurons were electroporated with 10 μg plasmids using the Amaxa Nucleofector II System (Lonza Amaxa, Germany) and cultured in neurobasal medium supplemented with B27 serum. Calcium phosphate transfection was used for neuronal morphological observations following the manufacturer’s instructions by preparing 4 μg plasmids mixed with 6 μl 2 M CaCl_2_ added into 2 × HBS.

### In Utero-electroporation

After anesthesia with sodium pentobarbital, pregnant mice at 14.5 days postcoitum were subjected to abdominal incision to expose the uterus. Mouse cortical progenitors were electroporated in vivo as described previously [46]. Animal care and experimental protocols were approved by the Institute of Mental Health, Peking University.

### Immunoblotting

Cortices and hippocampi of mice of indicated ages were lysed in ice-old RAPI buffer containing protease inhibitors (Roche). Proteins were electrophoresed on NUPAGE 4–12 % BT Gel (Invitrogen) and transferred to nitrocellulose membranes. Membranes were blocked with 5 % non-fat milk in PBS buffer for 1 h, incubated with primary antibodies at 4 °C overnight and with secondary antibodies for 1 h at room temperature after washing 3 times with PBST.

### Immunofluorescence

Dissociated neurons: Neurons were fixed with 4 % formaldehyde at 37 °C for 15 min. After blocking in BSA, neurons were incubated with the primary antibodies (4 °C, overnight).

Cortical sections: Brains were removed and fixed overnight in 4 % formaldehyde and thereafter transferred to 30 % sucrose/PBS (4 °C, overnight). Brains were embedded in OCT compound and sectioned in a cryostat. The 30 μm cryo-sections were incubated overnight at 4 °C with the primary antibodies.

### Antibodies

Primary antibodies used were rabbit anti-Gas7 (Sigma), rabbit anti-Cux1 (Santa Cruze), rabbit anti-GFP (Invitrogen), mouse anti-actin-mCherry (Sigma), rabbit anti-GAPDH (Cell Signaling Technology) and mouse anti-MYC (Santa Cruze) and alexa-546 phalloidin (Invitrogen).

Secondary antibodies included anti-rabbit IgG IRDye 680 and anti-mouse IgG IRDye 800 (LICOR Bioscience) for western blot and Alexa 488 anti-rabbit IgG, alexa 555 anti-rabbit IgG (Invitrogen) for immunostaining.

### SH-SY5Y cell culture, transfections, staining and filopodia measurements

SH-SY5Y cells were cultured in DMEM + 10 % FBS, plated in 3.5 cm dishes. Lipofectamine 2000 (5 μl) was mixed with 2 μg plasmids in Opti-MEM and added to cells for 4 h. Cells were cultured for 2 days, and then fixed. Cells were then washed 3 times in PBS, then blocked/permeabilized in 0.3 % triton + 4 % BSA (PBS) for 20 min. Cells were then incubated with GFP (Rabbit, 1:1000) and alexa-546 phalloidin (1:500) in PBS overnight.

To determine filopodia density, cells were imaged using an Olympus confocal microscope, 63×/1.4NA oil immersion objective. 5 × zoomed images were taken of representative cells expressing each construct. Images were then imported to NIH Image J.

### Animal behavioral tests

#### Weight

The Gas7-deficient mice were obtained from Prof. Sue Lin-Chao. Mice were fed and housed under 12-h light cycle. For behavioral experiments, we used hetero × hetero breeding, strict sex-matched controls and 2-month-old male mice. We recorded the weights of mice at 4, 6, 8, 10, 12 and 14 weeks of age.

#### Open field test

The size of the open field box was 40 × 40 × 40 cm, and the center zone was 40 % of total area. Mice were placed in the center of the chamber at the beginning of the assay, and mouse movements were recorded with a video camera for 60 min, and analyzed using the Ethovision 3.1 program (Noldus). In the first 10 min, we also analyzed the percentage of time that mice spent in the center and residual zones.

#### Dark-light transition test

An entrance enabled mice to freely move across the light and dark chambers. The size of both the light and dark chambers was 40 × 20 × 40 cm. Transition was defined as the translocation of all four feet of mice from one chamber to the other. Mice were allowed to explore the apparatus freely for 5 min. The time and distance spent in the dark and light chambers were measured.

#### Rotarod test

Mice were trained to run in the rotarod at the uniform acceleration speed of 4–40 rpm/min. For the test, the motor performance of mice was described as the time to fall recorded for each mouse. Animals were tested for three trials in a single day with an interval time of 30 min.

#### Morris water maze

Spatial learning and memory was assayed using the Morris water maze [47]. The pool was surrounded by contrasting shapes providing spatial cues. The escapes latencies, distance and swim speed were recorded by an automatic tracking system (Noldus). Animals were trained to locate a 10 cm diameter platform located within a 110 cm diameter pool of white tinted 22 °C water. First, mice were trained to swim for 60 s in the pool. Then training involved four trials from four different quadrants per day with 15 min intertrial interval, for 10 days. For the visible-platform maze, animals were trained for the first 2 days to locate an above-water platform with a visible cue. For the hidden-platform maze, mice were trained for the next 8 days to find an unmarked submerged platform. For reverse learning, the platform was placed in the opposite quadrant and mice were trained to find the hidden platform for 4 days. For the probe target test, the platform was removed and the time or distance in different quadrants was recorded during 60 s. If mice did not reach the platform within 60 s, they were placed on the platform for 17 s.

#### Pre-pulse inhibition

PPI was measured as described previously [48]. To measure startle, background was set to 66 dB. To calculate pre-pulse inhibition (% PPI), the startling pulse of 110 dB (white noise) was preceded (by 100 ms) by an average 15 ms (7–23 ms random order) nonstartling pre-pulse (70, 74 or 82 dB). PPI for a given pre-pulse intensity was calculated using the following formula: (100-(average startle response for pre-pulse trials/average startle response for pulse-only trials) × 100).

### Statistical analysis

The data from Fig. 1g and h are analyzed with Mann-Whitney *U* test and represented as the median, bar (interquartile range). Other data are represented as the mean ± SEM. Comparisons between two groups were made using Student’s unpaired two-tailed *t* tests. Comparisons among three or more groups were made using one-way ANOVA analyses followed by LSD multiple-comparisons test. Data marked with asterisks in the figures are significantly different from control as follows: *p < 0.05, **p < 0.01, ***p < 0.001.

### Ethics approval and consent to participate

This research was approved by the Ethics Committee of Institute of Mental Health, Peking University. All subjects provided written informed consent for the genetic study.

### Open Access

This article is distributed under the terms of the Creative Commons Attribution 4.0 International License (http://creativecommons.org/licenses/by/4.0/), which permits unrestricted use, distribution and reproduction in any medium, provided you give appropriate credit to the original author(s) and the source, provide a link to the Creative Commons license, and indicate if changes were made. The Creative Commons Public Domain Dedication waiver (http://creativecommons.org/publicdomain/zero/1.0/) applies to the data made available in this article, unless otherwise stated.

### Consent for publication

All authors have proved the manuscript and agreed with publication in *Molecular Brain*.

## Additional files

Additional file 1: Figure S1. The axon became the longest neurite at the stage 3. A At stage 3, the neuron was transfected with GFP and stained with an axon-specific marker Tau-1(red), Scale bar, 20 μm. (JPG 569 kb) Additional file 2: Figure S2. Regulation of Gas7 and △WW can impact development of neuron dendrites in vitro. A Representative images showing the cortical neurons transfected with truncates of Gas7, Gas7 and Mock by calcium phosphate at DIV 4, and observed at DIV10 and DIV21. Scale bars, 20 μm (up row) and 50 μm (below row) B The neurons transfected with Gas7 were also Emx1 (a specific neuronal marker, red) positive. Scale bar, 50 μm. C Representative images of cortical neurons transfected at DIV 4 for 3 d with Mock, ShB4 and shRNA-resistant Gas7 (Rescue) by calcium phosphate transfection. Scale bar, 20 μm. (JPG 2848 kb) Additional file 3: Figure S3. Gas7 induces filopodia formation in a F-BAR-dependent manner in SH-SY5Y cells. A-E Full-length Gas7 and truncates plasmids were transfected to SH-SY5Y cells. The cells expressed full-length Gas7 or other truncate protein and independently GFP (green), and were stained with phalloidin for F-actin (red). F Quantification of filopodia density as shown in Figure *S3A–5E*. Data represent mean ± SEM. *N* = 3 (6 neuron) for each group. Scale bar, 10 μm. (JPG 3469 kb) Additional file 4: Figure S4. The localization of Gas7 and F-actin. A The full-length Gas7 fused with MYC was transfected into neuron. The neuron expressing MYC (blue) and independently GFP (green) was stained with F-actin (red). Scale bars, 10 μm and 2 μm. (JPG 850 kb) Additional file 5: Figure S5. Overexpression of the truncated Gas7 △CC and △FCH showed normal neuron migration. A, B Representative images showing the E14.5 mouse cortices electroporated with overexpression of Gas7 ^△CC,^ Gas7^△FCH^ and pCAG-IRES vector (Mock) and examined at P0. The distribution of GFP-positive neurons is quantified in three zones. Data represent mean ± SEM. *N* = 4 for each group. Scale bar, 100 μm. (JPG 1783 kb) Additional file 6: Figure S6. Gas7-deficient mice showed normal locomoter activity, learning and memory. A Immunoblotting revealed the depletion of Gas7 in the cortex and hippocampus of the deficient mice. B–D Gas7-deficient mice performed normal in Morris water maze test. B Three genotype mice showed similar time to arrive at the visual platform. C Learning with the hidden platform and probe test with the platform removed. Latency to locate the hidden platform (up to 60s) showed no difference in three genotype mice. Three groups spend the similar time in the target quadrant during the probe test. D Reversal learning and reversal probe test. Mice showed no difference in learning the new location of the platform across the subsequent 4 d of reversal training (the platform was switched to the opposite quadrant). Three groups also spend similar time in the new target quadrant in reversal probe test. E Three genotype mice performed normal weight during 1 month age to adulthood. F, G Analysis of Open field locomoter activity level during 1 h and rotarod test among three groups. H, I Anxiety analysis including Dark-Light transition test and the first 10 min at open field test. Compared among three genotypes, there is no difference in anxiety level. *N* = 15 per genotype. (JPG 2751 kb)

## Abbreviations

BSA: bovine serum albumin
CI: confidence interval
DIV: days in vitro
F-actin: filamentous actin
GWAS: genome wide association study
Hetero: heterozygous
Homo: Gas7 deficient mice
MAF: minor allele frequency
OR: odds ratio
PSTPIP2: proline-serine-threonine phosphatase interacting protein 2
SNP: single nucleotide polymorphism
srGAP2: SLIT-ROBO Rho GTPase activating protein 2
SZ: schizophrenia
WAVE-1: protein SCAR1
WT: wild type
