# Supplementary figures and images for: Growth arrest specific gene 7 is associated with schizophrenia and regulates neuronal migration and morphogenesis
Source: Mol Brain. 2016 May 18;9:54. doi: 10.1186/s13041-016-0238-y (PMC4870797; doi:10.1186/s13041-016-0238-y)

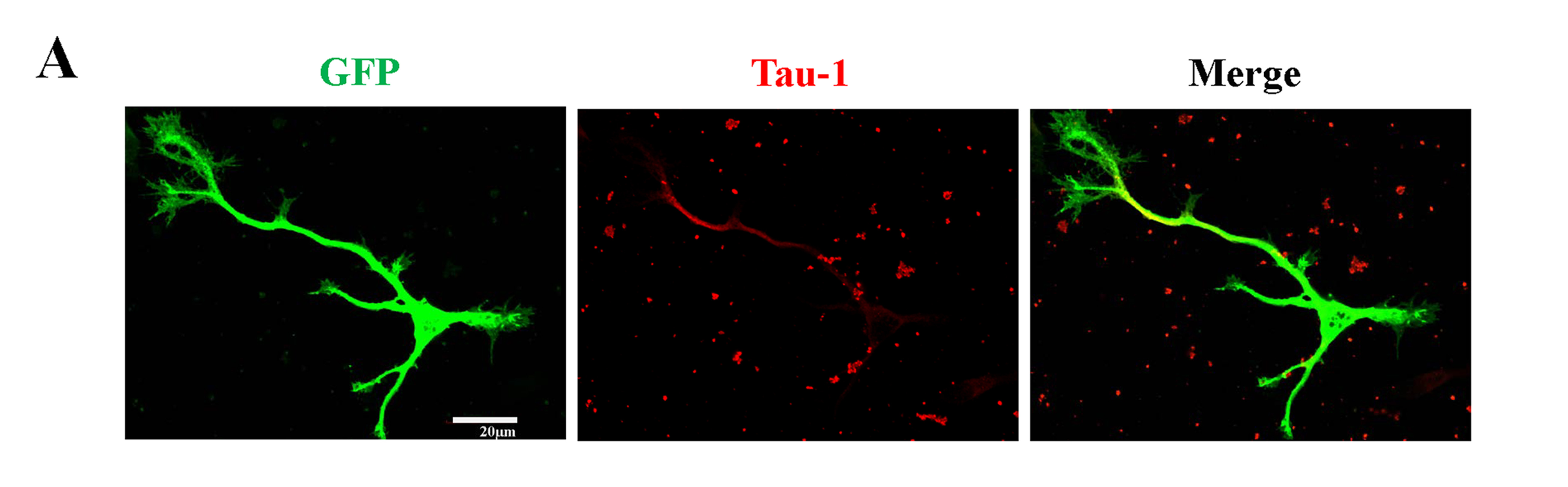

Supplement: Additional file 1: Figure S1. — The axon became the longest neurite at the stage 3. A At stage 3, the neuron was transfected with GFP and stained with an axon-specific marker Tau-1(red), Scale bar, 20 μm. (JPG 569 kb) [file 13041_2016_238_MOESM1_ESM.jpg]

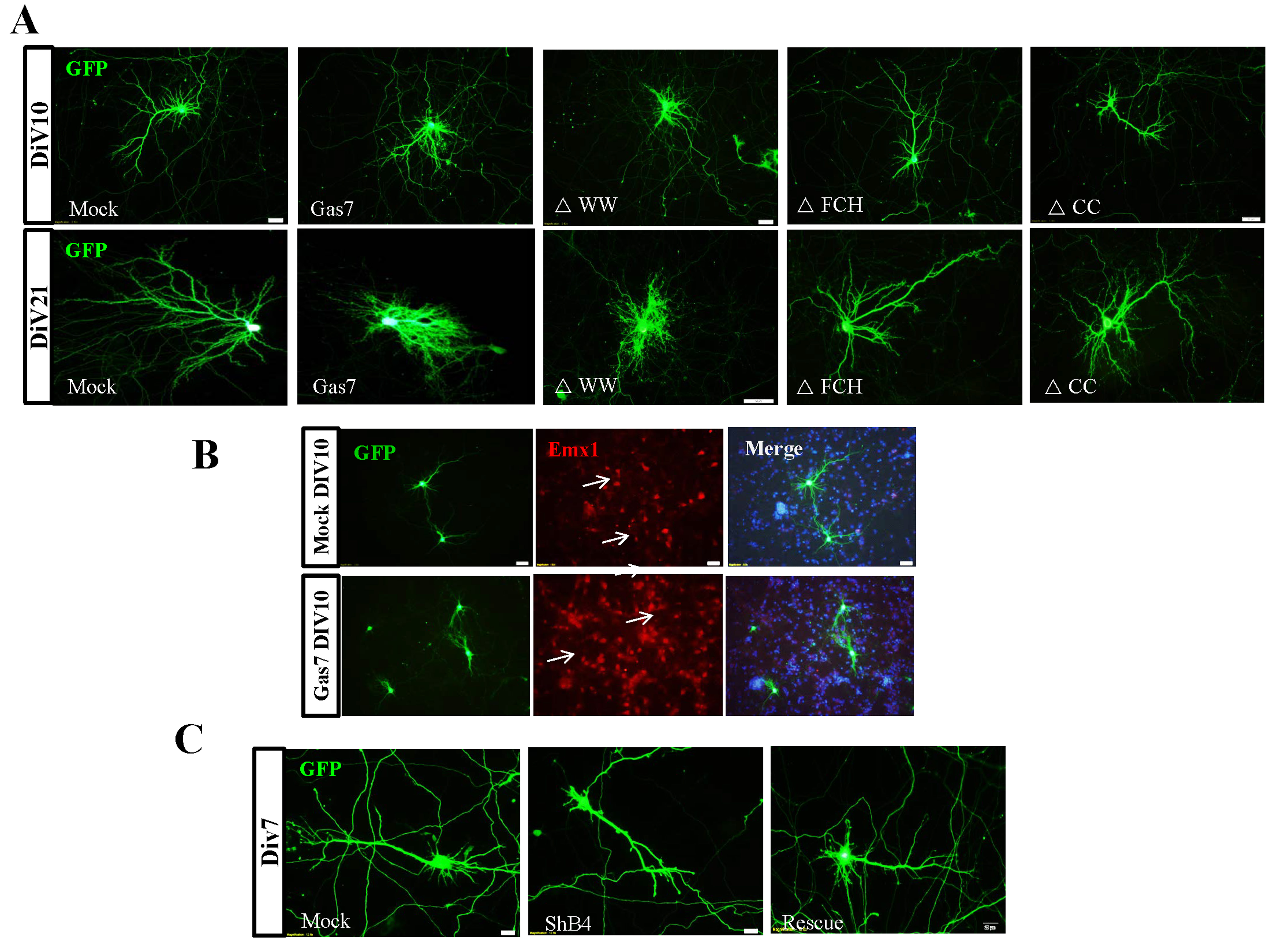

Supplement: Additional file 2: Figure S2. — Regulation of Gas7 and △WW can impact development of neuron dendrites in vitro. A Representative images showing the cortical neurons transfected with truncates of Gas7, Gas7 and Mock by calcium phosphate at DIV 4, and observed at DIV10 and DIV21. Scale bars, 20 μm (up row) and 50 μm (below row) B The neurons transfected with Gas7 were also Emx1 (a specific neuronal marker, red) positive. Scale bar, 50 μm. C Representative images of cortical neurons transfected at DIV 4 for 3 d with Mock, ShB4 and shRNA-resistant Gas7 (Rescue) by calcium phosphate transfection. Scale bar, 20 μm. (JPG 2848 kb) [file 13041_2016_238_MOESM2_ESM.jpg]

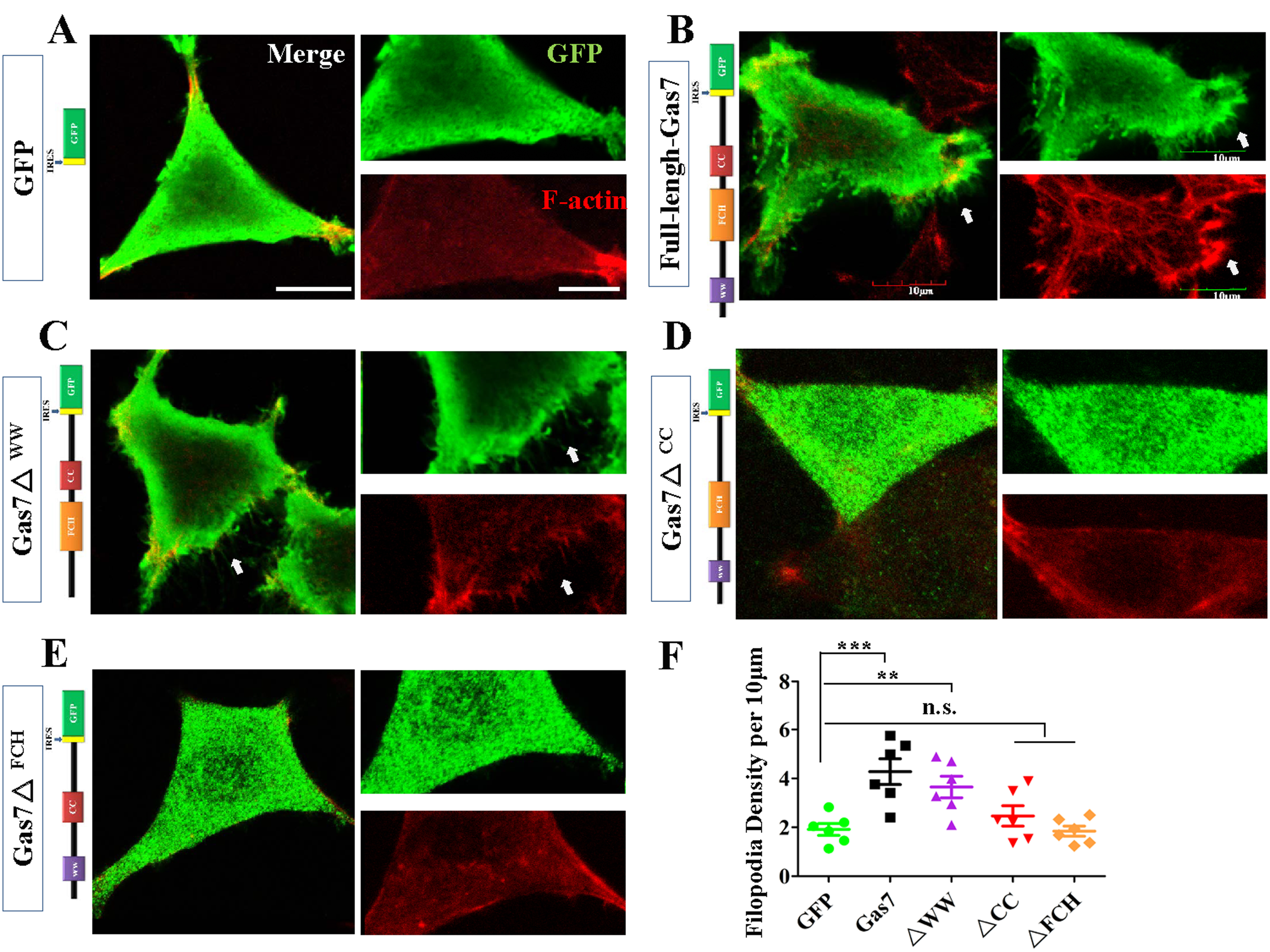

Supplement: Additional file 3: Figure S3. — Gas7 induces filopodia formation in a F-BAR-dependent manner in SH-SY5Y cells. A-E Full-length Gas7 and truncates plasmids were transfected to SH-SY5Y cells. The cells expressed full-length Gas7 or other truncate protein and independently GFP (green), and were stained with phalloidin for F-actin (red). F Quantification of filopodia density as shown in Figure S3A–5E. Data represent mean ± SEM. N = 3 (6 neuron) for each group. Scale bar, 10 μm. (JPG 3469 kb) [file 13041_2016_238_MOESM3_ESM.jpg]

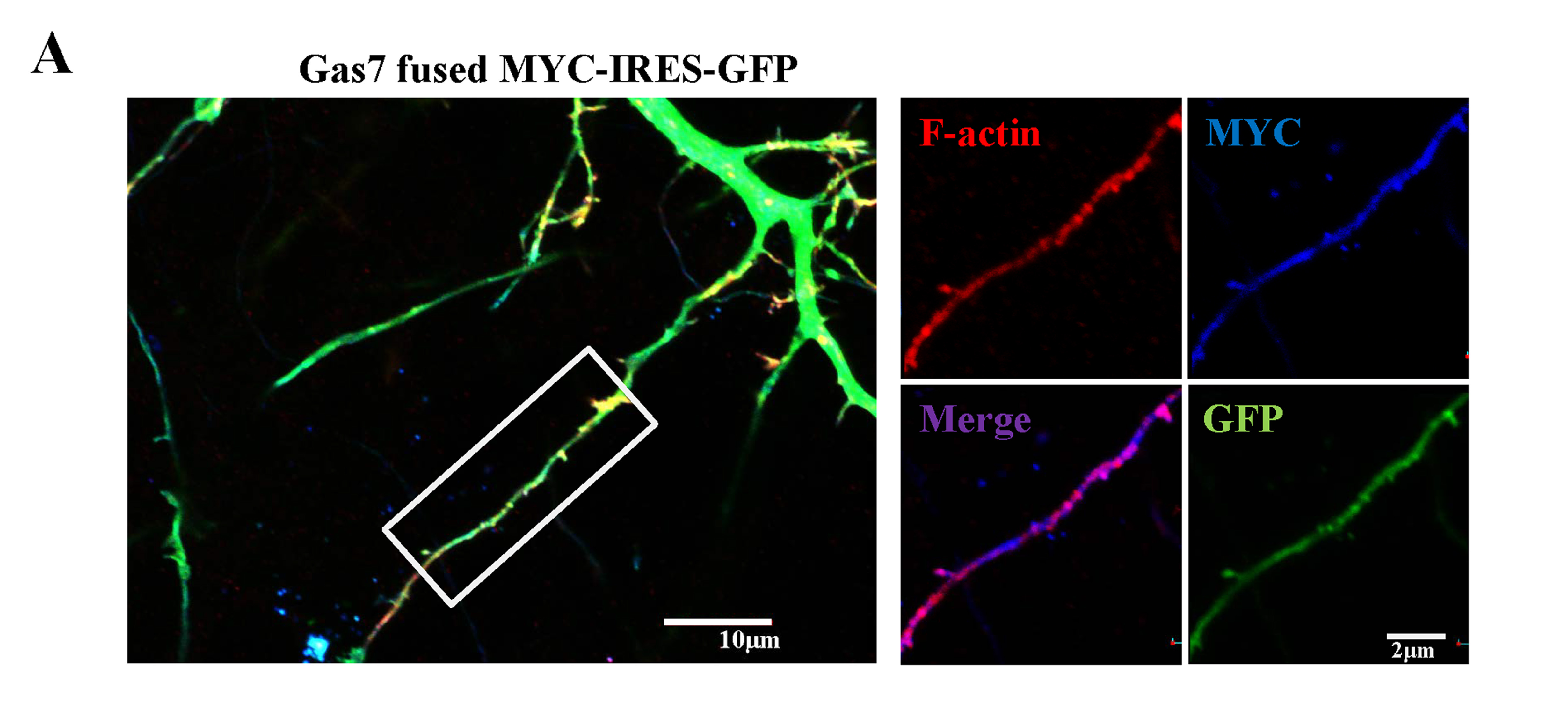

Supplement: Additional file 4: Figure S4. — The localization of Gas7 and F-actin. A The full-length Gas7 fused with MYC was transfected into neuron. The neuron expressing MYC (blue) and independently GFP (green) was stained with F-actin (red). Scale bars, 10 μm and 2 μm. (JPG 850 kb) [file 13041_2016_238_MOESM4_ESM.jpg]

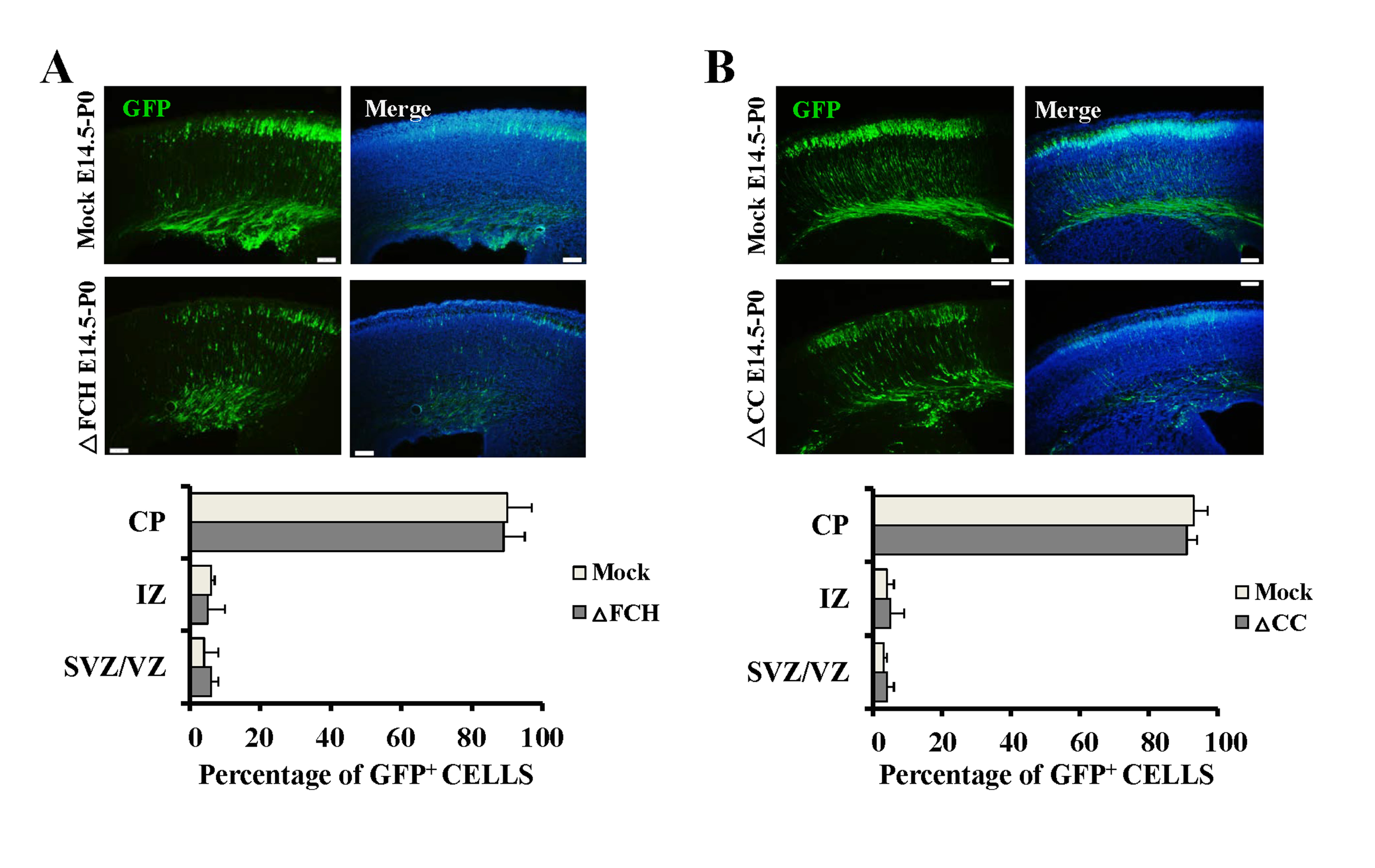

Supplement: Additional file 5: Figure S5. — Overexpression of the truncated Gas7 △CC and △FCH showed normal neuron migration. A, B Representative images showing the E14.5 mouse cortices electroporated with overexpression of Gas7 △CC, Gas7△FCH and pCAG-IRES vector (Mock) and examined at P0. The distribution of GFP-positive neurons is quantified in three zones. Data represent mean ± SEM. N = 4 for each group. Scale bar, 100 μm. (JPG 1783 kb) [file 13041_2016_238_MOESM5_ESM.jpg]

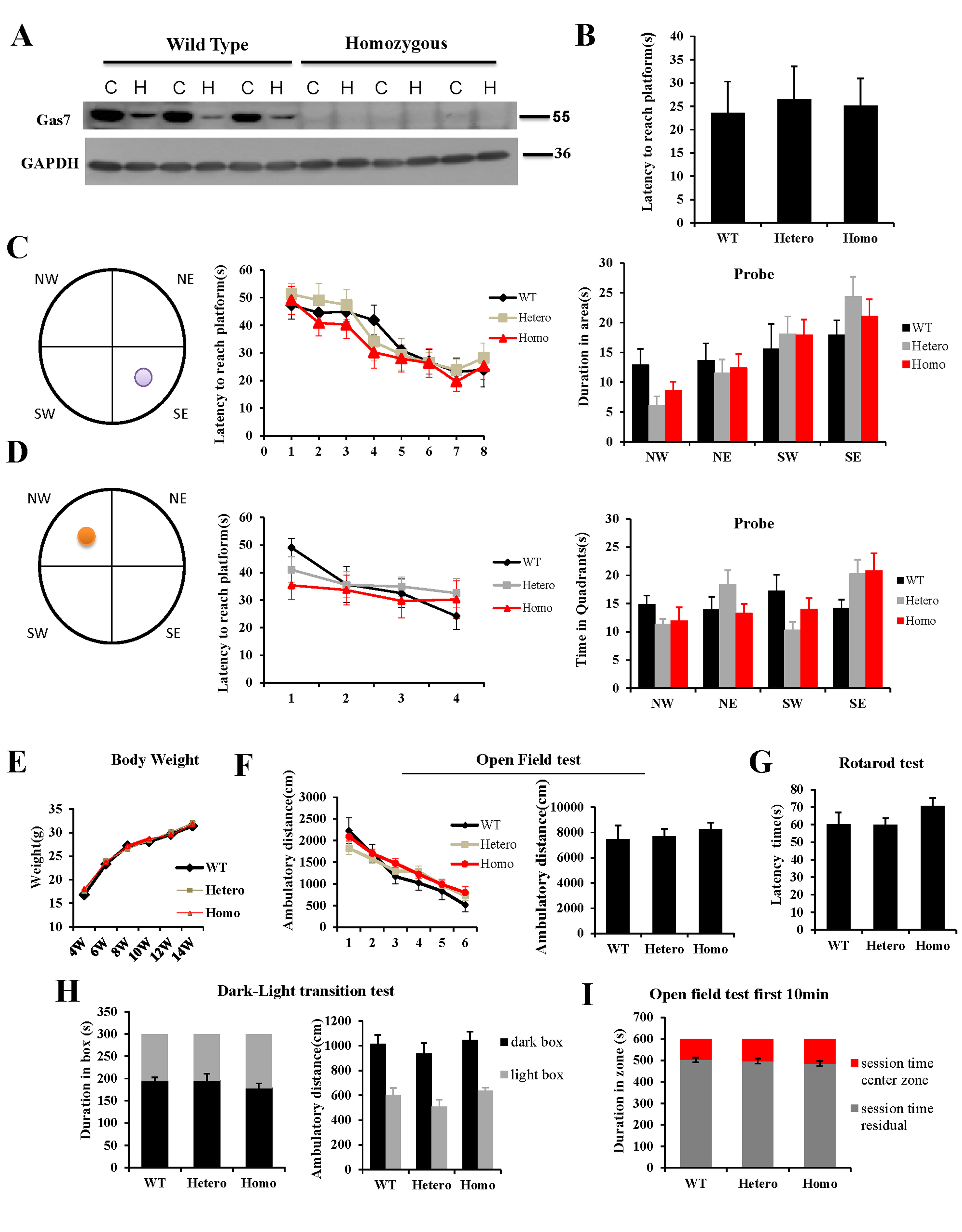

Supplement: Additional file 6: Figure S6. — Gas7-deficient mice showed normal locomoter activity, learning and memory. A Immunoblotting revealed the depletion of Gas7 in the cortex and hippocampus of the deficient mice. B–D Gas7-deficient mice performed normal in Morris water maze test. B Three genotype mice showed similar time to arrive at the visual platform. C Learning with the hidden platform and probe test with the platform removed. Latency to locate the hidden platform (up to 60s) showed no difference in three genotype mice. Three groups spend the similar time in the target quadrant during the probe test. D Reversal learning and reversal probe test. Mice showed no difference in learning the new location of the platform across the subsequent 4 d of reversal training (the platform was switched to the opposite quadrant). Three groups also spend similar time in the new target quadrant in reversal probe test. E Three genotype mice performed normal weight during 1 month age to adulthood. F, G Analysis of Open field locomoter activity level during 1 h and rotarod test among three groups. H, I Anxiety analysis including Dark-Light transition test and the first 10 min at open field test. Compared among three genotypes, there is no difference in anxiety level. N = 15 per genotype. (JPG 2751 kb) [file 13041_2016_238_MOESM6_ESM.jpg]
